# Breadth and magnitude of antigen-specific antibody responses in the control of plasma viremia in simian immunodeficiency virus infected macaques

**DOI:** 10.1186/s12985-016-0652-x

**Published:** 2016-12-01

**Authors:** Bapi Pahar, Carys S. Kenway-Lynch, Preston Marx, Sudesh K. Srivastav, Celia LaBranche, David C. Montefiori, Arpita Das

**Affiliations:** 1 Division of Comparative Pathology, Tulane National Primate Research Center, 18703 Three Rivers Road, Covington, LA 70433 USA; 2 Tulane University School of Medicine, New Orleans, 70112 LA USA; 3 Division of Microbiology, Tulane National Primate Research Center, Covington, 70433 LA USA; 4 Department of Biostatistics and Bioinformatics, Tulane University, New Orleans, 70112 LA USA; 5 Department of Surgery, Duke University School of Medicine, Durham, NC 27710 USA

**Keywords:** Antibody, Breadth, Correlate of protection, Neutralizing antibodies, Peptides, Rhesus macaque, SIV, SIV-antigens

## Abstract

**Background:**

Increasing evidence suggests an unexpected potential for non-neutralizing antibodies to prevent HIV infection. Consequently, identification of functional linear B-cell epitopes for HIV are important for developing preventative and therapeutic strategies. We therefore explored the role of antigen-specific immune responses in controlling plasma viremia in SIV infected rhesus macaques.

**Methods:**

Thirteen rhesus macaques were inoculated either intravaginally or intrarectally with SIV_MAC_251. Peripheral blood CD4+ T-cells were quantified. Plasma was examined for viremia, antigen specific IgG, IgA and IgM binding responses and neutralizing antibodies. Regions containing binding epitopes for antigen-specific IgG, IgM and IgA responses were determined, and the minimum size of linear Envelope epitope responsible for binding antibodies was identified.

**Results:**

The presence of neutralizing antibodies did not correlate the outcome of the disease. In a few SIV-infected macaques, antigen-specific IgG and IgM responses in plasma correlated with decreased plasma viremia. Early induction and the breadth of antigen-specific IgG responses were found to be significantly correlated with the control of plasma viral load. Immunoglobulin classes share similar functional linear B-cell epitopes. SIV-specific linear envelope B-cell epitopes were found to be 12 amino-acids in length.

**Conclusions:**

Early induction of combination of peptide-specific IgG responses were found to be responsible for the control of plasma viral load and indicative of disease outcome in SIV-infected rhesus macaques and might be important for the development of therapeutic strategies for control or prevention of HIV/AIDS.

**Electronic supplementary material:**

The online version of this article (doi:10.1186/s12985-016-0652-x) contains supplementary material, which is available to authorized users.

## Background

HIV-1 infection is associated with polyclonal B-cell activation, hypergammaglobulinemia, the presence of immature/transitional CD10+ or exhausted CD27 negative B-cells in blood [1, 2], exhaustion of tissue-like memory (CD20(hi)/CD27(−)/CD21(lo)) B-cells [3], loss of total B-cell populations [4, 5], and nonspecific switching from IgM to IgG, IgA and IgE responses. Our recent data demonstrated defective memory (CD21 + CD27+) B-cell proliferation in selective tissues in simian immunodeficiency virus (SIV)-infected macaques [6, 7]. Therefore, the maintenance of normal and effective humoral immune responses may be the key to the prevention and control of HIV/SIV infection. Recent reports emphasize that HIV-specific antibodies (Abs), instead of T-cell responses, may correlate better with protection in seronegative partners of HIV-1 infected individuals [8]. Moreover, other emerging studies demonstrate a correlation between anti-HIV antibodies and protection from infection, although these protective Abs are not strictly neutralizing in vitro [9]. Furthermore, lymphocytic choriomeningitis virus infection in the mouse model has also shown that non-neutralizing Abs elicited early in infection are capable of binding to the virus and limiting it's spread [10].

B-cell epitopes are described either as conformational (discontinuous, assembled) epitopes where multiple discontinuous amino-acids (aa) segments are folded to produce a unique conformational epitope complementary to the antibody, termed a contact epitope [11–14], or linear epitopes (continuous, sequential) where epitopes do not incorporate protein folding and can be represented by linear peptide sequence [15]. The continuous maturation of the immune response following SIV infection emphasizes the need to study the generation of SIV-specific Ab responses, antigen-antibody binding efficacy, and their potential importance in regulating disease progression.

The present study was designed to determine the importance of total immunoglobulin, antigen-specific immunoglobulin responses against whole viral lysate (WVL), peptides corresponding to Env, Gag, Nef, and Tat and neutralizing antibodies (NAbs) in controlling plasma viral load (pVL) in SIV_MAC_251 infected rhesus macaques (RMs). Regions containing binding epitopes for antigen-specific IgG, IgM and IgA responses during different stages of SIV infection were determined, and the minimum size of linear Env epitope responsible for binding Abs was identified. Our findings suggest that conformational IgG and IgM responses as well as breadth of different peptide-specific functional IgG responses are indicative of disease outcome. The presence of NAbs against neutralization-sensitive and resistant pseudovirus did not predict the outcome of the disease.

## Methods

### Animals

All animals in this study were housed at the Tulane National Primate Research Center (TNPRC) in accordance with the standards incorporated in the Guide for the Care and Use of Laboratory Animals [16]. All of TNPRC animal housing meets the Laboratory and Animal Biosafety Level 2+ requirements recommended for hepatitis, AIDS, and other viral agents related studies in the CDC/NIH publication “Biosafety in Microbiological and Biomedical Laboratories”. The Tulane Institutional Animal Care and Use Committee (IACUC) of the TNPRC approved all animal procedures related to this manuscript. The TNPRC is fully accredited by the Association for the Assessment and Accreditation of Laboratory Animal Care (Animal Welfare Assurance A-4499-01). Virus inoculation, sample collections from animals were performed under the direction of veterinarians. Every effort was made to avoid unnecessary discomfort and pain to animals. At the TNPRC, animal discomfort or pain was alleviated by appropriate use of anesthetic medications. Rhesus macaques were sedated with ketamine (10 mg/kg body weight) whenever they were removed from their home cage for routine blood collection, physical examinations or other minor procedures. Animals were euthanized humanely using the standard method of euthanasia for nonhuman primates, where animals were euthanized using Telazol and buprenorphine followed by a lethal intravenous dose of sodium pentobarbital. This method was consistent with the recommendation of the American Veterinary Medical Association Guidelines.

### Animal and tissue sampling

Thirteen adult Indian RMs (*Macaca mulatta*), initially negative for SIV, HIV-2, type D retrovirus and simian T-cell leukemia virus 1 infection were inoculated with 300–500 TCID_50_ of SIV_MAC_251-CX intravaginally (Ivag) or intrarectally (IR) (Table 1). Two out of 13 RMs were positive for Mamu-A*01 and majority were negative for Mamu-B*17 alleles. Mamu-A*01 and Mamu-B*17 alleles were found to be linked with lower viral set points and slow disease progression in SIV infected RMs [17–20]. However, the independent presence of Mamu-B*17 allele does not predict the outcome of SIV infection [21]. Intravaginally inoculated female macaques were treated with depot medroxyprogesterone acetate (30 mg intramuscularly) 28d prior to SIV exposure [22]. Sodium heparin and EDTA peripheral blood (PB) were collected at sequential time points for analysis described below.

**Table 1:** List of adult Indian rhesus macaques examined

Route	Animal Number	Age (Years)	Sex ^a^	SIV_MAC_251 Dosage (TCID_50_^b^)	Mamu-A*01 Allele	Mamu B*17 Allele	Progressor	Major Pathological Findings
Intravaginal (Ivag)	BC35	12.0	F	300	-	ND	-	-
	CL86	10.4	F	500	-	ND	+	Severe lymphoid hyperplasia and dysplasia in spleen, all lymph nodes, tonsil, and many other organs; Mild intestinal pneumonia
	CL87	10.0	F	300	-	ND	-	-
	DE50	9.4	F	500	-	-	+	Generalized amyloidosis and lymphoid hyperplasia
	FK88	5.9	F	500	-	-	+	Mild to moderate lymphoid hyperplasia and dysplasia in spleen, multiple lymph nodes and organs
	GN91	3.8	F	500	-	-	-	-
Intrarectal (IR)	AE14	8.0	M	500	-	-	+	Amyloid deposits in the liver; small number of pneumocystis and SIV giant cells in lung; Cryptosporidium infection in small intestine
	AP09	7.3	M	500	+	-	-	-
	AP64	7.0	M	500	-	-	-	-
	BG21	6.3	M	500	-	-	-	-
	N107	12.4	M	500	-	-	-	-
	P205	11.4	F	500	-	-	+	Amyloidosis in the small intestine and enterocolitis; lesions in lung are typical of viral pneumonia with presence of SIV giant cells
	T153	9.5	F	500	+	-	-	-

^a^F and M denote female and male respectively; ^b^TCID_50_: Tissue culture infectivity dose at 50%; ND: not done; “+” and “-“ denotes positive and negative results respectively for the respective column

### Plasma viral load quantification

Plasma viral RNA was quantified either by bDNA signal amplification assay for IR macaques (Siemens Diagnostics, USA) [22, 23] or by quantitative RT-PCR assay for Ivag macaques (Wisconsin National Primate Research Center Virology Core laboratory) [24, 25]. The lower limit of RNA detection for bDNA and RT-PCR assays were 125 and 60 SIV-RNA copies/ml of plasma respectively.

### Peripheral blood T-cell immunophenotyping

PB T-cell immunophenotyping was performed using anti-CD3-FITC (SP34-2), anti-CD4-APC (L200) and anti-CD8-PE (RPA-T8) monoclonal antibodies (MAbs) obtained from BD Biosciences as reported earlier [5, 26]. At least 20,000 events were collected by gating on lymphocytes. Data were acquired in a BD LSRII flow cytometer within 24 h after staining and analyzed further using FlowJo software (TreeStar Inc.) [5, 27]. Absolute count of CD4+ T-cell population was calculated using flow cytometry and CBC data.

### Neutralization assay

Neutralizing antibody (NAb) activity was measured in 96-well culture plates by using Tat-regulated luciferase (Luc) reporter gene expression to quantify reductions in virus infection in TZM-bl cells against the neutralization-sensitive SIV_MAC_251.6 and the neutralization-resistant SIV_MAC_251.30 pseudoviruses, using MLV-pseudotyped virus as a negative control for non-specific virus inhibition as described previously [28]. TZM-bl cells were obtained from the NIH AIDS Research and Reference Reagent Program, as contributed by John Kappes and Xiaoyun Wu. Heat inactivated plasma samples were diluted over a range of 1:20 to 1:43740 in cell culture medium and pre-incubated with virus (~150,000 relative light unit equivalents) for 1 h at 37 ºC before addition of cells. Following a 48 h incubation, cells were lysed and Luc activity determined using a microtiter plate luminometer and BriteLite Plus Reagent (Perkin Elmer). Neutralization titers are the sample dilution at which relative luminescence units (RLU) were reduced by 50% compared to RLU in virus control wells after subtraction of background RLU in cell control wells.

### Measurement of antigen-specific and total immunoglobulin responses

Antigen-specific IgG, IgA and IgM were detected in plasma using ELISA as previously described [5, 29, 30]. Purified SIV_MAC_251 WVL was used as coating antigen (5 μg/ml, Applied Biosystems) to quantify antibody responses against conformational/discontinuous epitopes. Total IgG, IgM and IgA concentrations were measured by ELISA as previously described [5, 7], where plates were coated with either anti-monkey IgG Fc/7s (Accurate Chemicals), IgM Fc (Accurate Chemicals) or IgA Fc (Alpha Diagnostic International) Abs. All samples were assayed in duplicate with appropriate positive and negative controls. For quantification of WVL-specific and total immunoglobulin responses, rhesus IgG, IgA (NIH-Nonhuman Primate Reagent Resource) and IgM (Fitzgerald Industries International) standards were used. Nonlinear regression using a sigmoidal dose-response variable slope model was used to interpolate concentrations from the standard curve. For WVL-specific Ig responses, positive values had to exceed the mean + 2SD of all animal’s pre-infection readings for a specific antigen at absorbance 490 nm. To increase stringency and to account for variation between animals, the absorbance values for each individual animal also had to exceed two times the value for the specific animal prior to infection to be classified as positive.

### Measurement of peptide-specific immunoglobulin responses

SIV-Env (catalog- 6883), SIV-Gag (catalog- 6204), SIV-Nef (catalog- 8762), and SIV-Tat (catalog- 6207) 15-mers with 11-aa overlap peptides (NIH AIDS Research and Reference Reagent Program) were used as coating antigen. Plates were coated with peptide pools (PPs, 4–10 peptides per pool, 5 μg/ml of each peptide in 0.1 M sodium carbonate monohydrate, pH 9.6) to determine antibody responses generated against linear/continuous epitopes by ELISA as described earlier [5, 29, 30]. For all peptide-specific ELISA values, the positive responses were determined as specified above for WVL-specific responses. Cumulative OD values were calculated by the summation of OD/490 values for each positive peptide pool, after subtraction of the pre-infection value for each peptide pool in a protein.

### Statistical analysis

Statistical significance for immunoglobulin quantitation data was determined using a one-way ANOVA. The Bonferroni method was used as a *post hoc* multiple comparison test for all means. Statistical differences between groups were tested using a two-tailed unpaired t-test. Pearson coefficient of determination analysis was performed to calculate correlation between pVLs and immunoglobulin responses and between pVLs and breadth of antigen-specific antibody responses using SAS software (version 9 in a windows environment). For all analyses, P values <0.05 were considered significant.

## Results

### Plasma viral load and peripheral CD4+ count demonstrate the progression of SIV infection in SIV-infected macaques

**Fig. 1:**
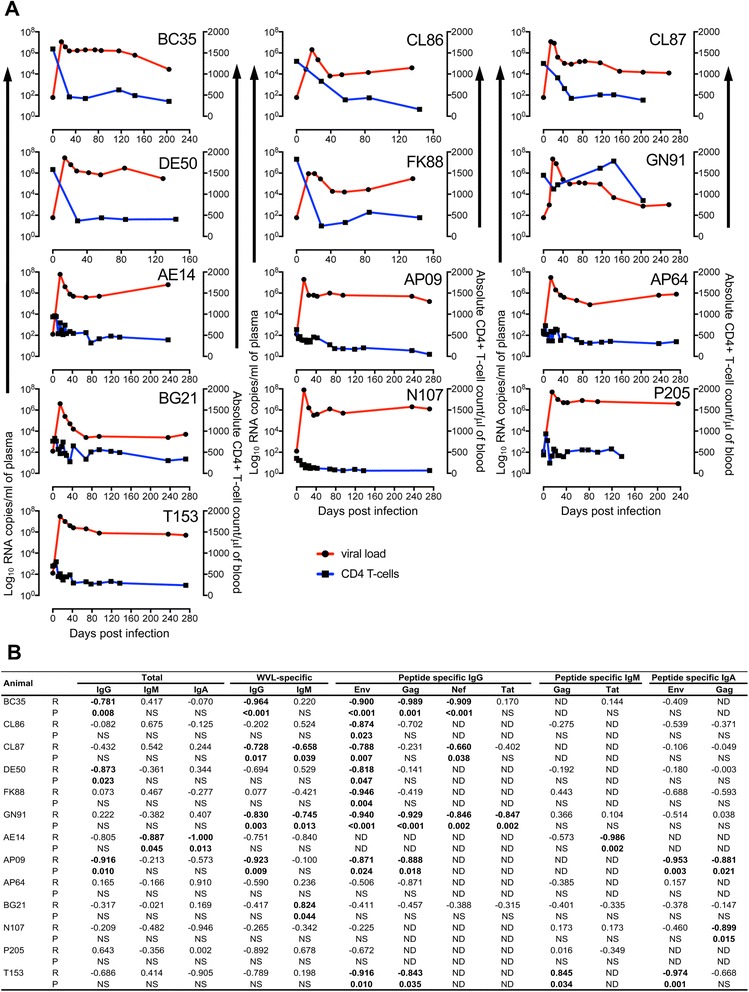
**a** Plasma viral load and absolute CD4+ T-lymphocyte counts over the course of 257 days for intravaginally (Ivag, *n* = 6, upper rows) and 272 days for intrarectally (IR, *n* = 7, bottom rows) SIV_MAC_251 inoculated macaques were shown. One macaques, GN91 was able to maintain plasma viral load to approximately 1 × 10^3^ RNA copies/ml of plasma following the initial peak of infection. The remaining macaques retained a high viral load throughout infection amongst which CL86, DE50, FK88, AE14 and P205 progressed to AIDS rapidly and were euthanized accordingly. Note GN91 was able to preserve peripheral CD4+ T-cell population after SIV infection for upto 150dpi. **b** Pearson coefficient of determination analysis between plasma viral load and either total immunoglobulins or antigen specific immunoglobulins were shown for all macaques following SIV_MAC_251 inoculation for all time points. No significant correlation between plasma viral load and total or antigen-specific immunoglobulin responses were detected in two macaques (AP64 and P205) for all time specified above. Additionally, no significant correlations were found for WVL-specific IgA or peptide-specific IgM for Env and Nef. No Tat or Nef-specific IgA responses were detected. R and P denote Pearson R and probability values respectively for each row when correlated with plasma viral load. NS denotes non-significant P-value (*p* > 0.05). ND denotes a correlation cannot be calculated. Significant correlation values were shown in bold numbers

Male to female/female to male and male to male sexual transmission via intravaginal and intrarectal route respectively are responsible for 75–85% of the HIV transmission [31]. In this study, we have used two groups of animals inoculated via Ivag (represents male to female transmission) or IR (represents male to male transmission) route to determine whether the inoculation route had any effect in regulating antigen-specific antibody and neutralizing antibody responses. We were also interested to determine whether the inoculation route had any impact on the breadth of immune responses. In all SIV-infected RMs, peak plasma viral replication (log_10_ 6.3–7.8 RNA copies/ml of plasma) was detected between 14 and 21 day post infection (dpi, Fig. 1a). Only one animal GN91 was able to control pVL to approximately 1 × 10^3^ RNA copies/ml of plasma following the initial peak of viremia (Fig. 1), whereas the remaining animals had plasma SIV-RNA greater than 10,000 copies/ml of plasma. Several of these animals (CL86, DE50, FK88, AE14, and P205) progressed to AIDS rapidly and were euthanized accordingly (Table 1). There were no difference in the mean viral set points (measured 25 to 95dpi) between Ivag and IR infected RMs.

Following SIV_MAC_251 infection, absolute CD4 count in peripheral blood decreased rapidly in the majority of the animals except GN91, in which CD4+ T-cell count decreased after 150dpi (Fig. 1a). Animal N107 had a low peripheral CD4+ T-cell count from the beginning compared to other macaques and by 18dpi the CD4+ T-cell count was below 200 cells/μl of blood and remained low throughout this study. The absolute CD4+ T-cell counts do not completely reflect the status of pVL as AE14 and BG21 had similar patterns of CD4+ T-cell count in peripheral blood, however the plasma viral load in AE14 remained 2.2 log higher than the viral load in BG21 from 68dpi onwards.

### Neutralizing antibody titers did not predict the disease outcome or the progression of disease

All thirteen RMs were selected to perform NAb assays at several different time points after SIV infection (Table 2). Neutralization of the highly neutralization-sensitive Tier 1A virus, SIVmac251.6 was detectable in all animals by 25–27 days post-infection and rose to very high titers over the time course of the samples received. Out of a total of 13 RMs, neutralization of SIVmac251.30, which exhibits an insensitive Tier 3 neutralization phenotype that more closely approximates the infecting virus, was detected at 42 and/or 95dpi in 4 RMs and at 257dpi in 1 RM. A few of the titers were fairly high in RMs AE14, AP09, CL87 and P205. However, AE14 and P205 progressed to AIDS rapidly despite the presence of NAb titers against neutralization-resistant SIVmac251.30 pseudovirus (Table 2). We did not observe any difference in Nab titers between Ivag and IR infected RMs.

**Table 2:** Neutralizing antibody titers in SIV_MAC_251 infected macaques

Route	Animal Number	Days post SIV infection	ID_50_ in TZM-bl cells	
			SIV_MAC_251.6	SIV_MAC_251.30
Intravaginal (Ivag)	BC35	0	-	-
		27	6350	-
		41	>43740	-
		84	>43740	-
		257	>43740	-
	CL86	0	-	-
		27	>43740	-
		41	>43740	-
		84	>43740	-
		126	>43740	-
	CL87	0	-	-
		27	21470	-
		41	>43740	-
		84	>43740	-
		257	>43740	313
	DE50	0	-	-
		27	>43740	-
		41	>43740	-
		84	>43740	-
		136	>43740	-
	FK88	0	-	-
		27	>43740	-
		41	>43740	-
		84	>43740	-
		136	>43740	-
	GN91	0	-	-
		27	7091	-
		41	>43740	-
		84	>43740	-
		257	>43740	-
Intrarectal (IR)	AE14	0	-	-
		25	736	-
		42	16076	214
		95	11601	-
		236	3323	-
	AP09	0	-	-
		25	4988	-
		42	>43740	707
		95	>43740	1000
		236	>43740	-
	AP64	0	-	-
		25	>43740	-
		42	>43740	-
		95	>43740	-
		236	>43740	-
	BG21	0	-	-
		25	>43740	-
		42	>43740	-
		95	>43740	-
		236	>43740	-
	N107	0	-	-
		25	8981	-
		42	>43740	-
		95	>43740	-
		236	>43740	-
	P205	0	-	-
		25	4465	-
		42	7942	93
		95	27282	285
		236	>43740	-
	T153	0	-	-
		25	2359	-
		42	>43740	-
		95	>43740	24
		236	>43740	-

Note: “-” denotes no positive responses detected for that specific time point

### In a few SIV-infected macaques, antigen–specific IgG and IgM responses in plasma correlated with decreased plasma viremia

**Fig. 2:**
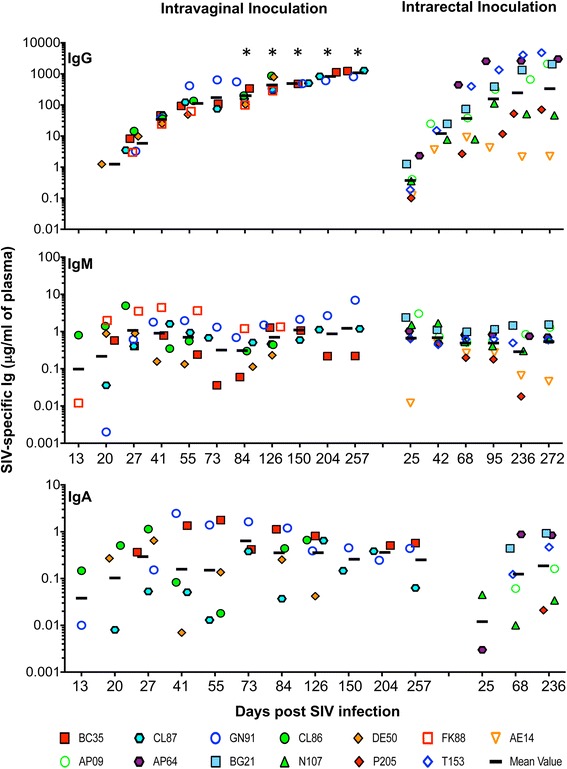
Anti-SIV immunoglobulin responses in plasma during SIV_MAC_251 infection. Whole viral lysate (WVL)-specific immunoglobulin concentrations was calculated for each antibody isotypes at different time points of SIV_MAC_251 infection in both intravaginally (*n* = 6) and intrarectally (*n* = 7) inoculated animals. The geometric mean values of the respective responses were shown by the line for the specific time point in each plot. Asterisks indicate statistically significant differences (*p* < 0.05) when compared to 13dpi

Total and WVL-specific IgG, IgM and IgA responses were measured in both IVag and IR inoculated SIV infected macaques (Fig. 2 and Additional file 1: Table S1). Total IgG concentration remained largely consistent (IVag: 2989–9367 μg/ml; IR: 1533–4028 μg/ml) while SIV-specific IgG responses increased significantly during the course of SIV infection in both IVag and IR infected macaques (geometric mean values 1084 μg/ml at 257 days post infection (dpi) and 334 μg/ml at 272dpi in IVag and IR infection respectively; Fig. 2). Increased SIV-specific IgG responses in chronic infection correspond with increased peptide-specific IgG responses in both groups. Total IgM concentration also did not vary significantly throughout the course of infection (IVag: 550–1777 μg/ml; IR: 1503–3393 μg/ml; Additional file 1: Table S1). However, SIV infected macaques showed an early peak in SIV-specific IgM concentrations in acute infection (Ivag: geometric mean value 1.1 μg/ml at 27dpi; IR: mean value 0.7 μg/ml at 25dpi; Fig. 2) that decreased at later time points. SIV-specific IgM concentrations were lower in animals infected via the IR route, however, the trend between the two inoculation routes remained same. Total IgA responses varied non-significantly throughout infection (IVag: 632–1388 μg/ml; IR: 797–1093 μg/ml; Additional file 1: Table S1). However, SIV-specific IgA responses were detected in 5 out of 6 Ivag infected RMs and the level peaked at 73dpi. AE14 from IR infected RMs did not generate WVL-specific IgA responses and the overall IgA titer in rest of the IR infected animals was lower than Ivag infected RMs (Fig. 2).

Pearson coefficient of determination analysis was performed to determine correlation between pVLs and immunoglobulin responses (20dpi to 257dpi and 25dpi to 272dpi for Ivag and IR RMs respectively, Fig. 1b) as reported earlier [32]. Total Ab responses in four (BC35, DE50, AE14 and AP09) out of thirteen macaques were negatively correlated with pVLs suggesting that total IgG, IgM or IgA had minimal role in controlling pVL (Fig. 1b). Similarly WVL-specific Ab responses in four (BC35, CL87, GN91 and AP09) out of thirteen RMs were negatively correlated with pVLs suggested that WVL-specific IgG or IgM also had minimal role in controlling pVL (Fig. 1b).

### Combination of different peptide-specific IgG responses found to correlate with the control of plasma viral load

**Fig. 3:**
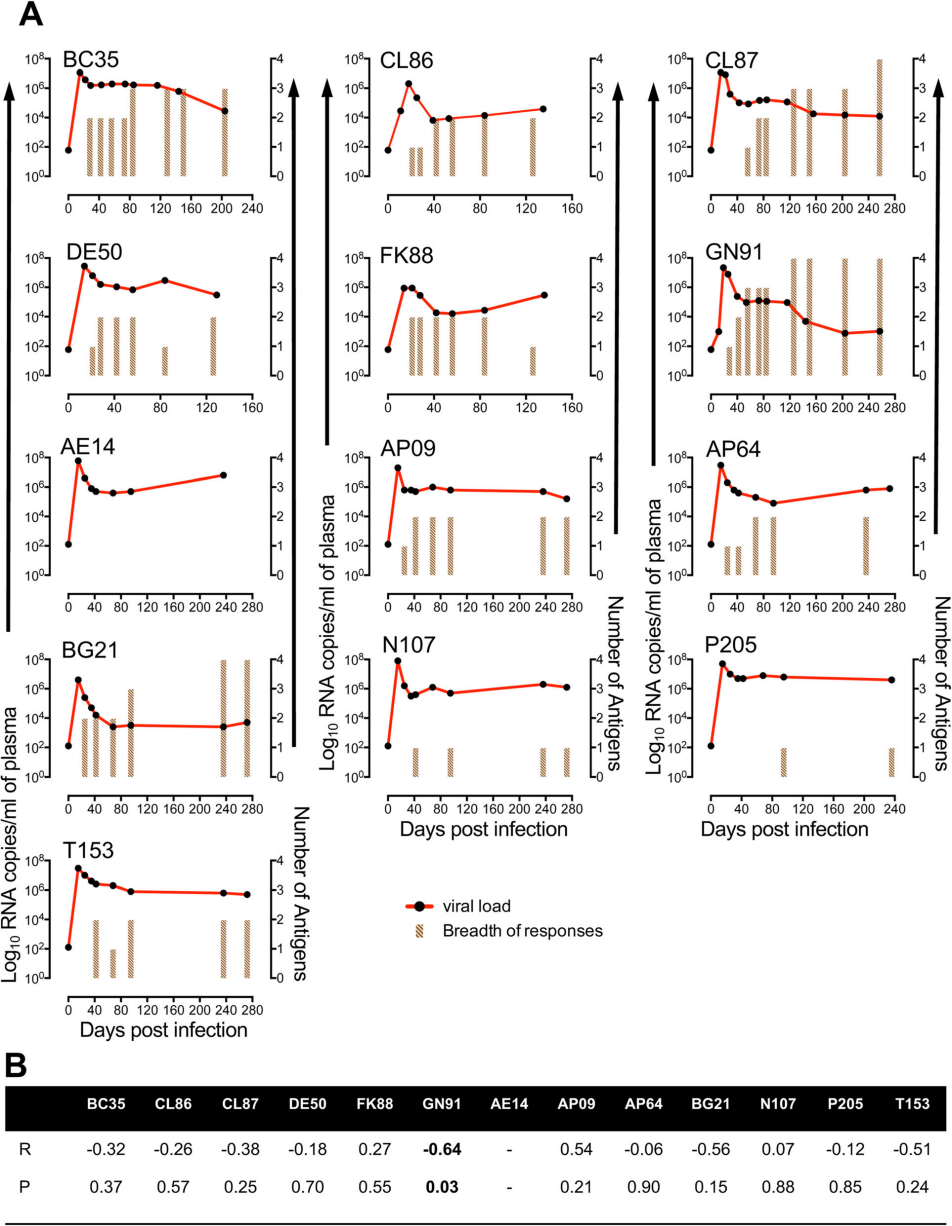
**a** Plasma viral load and number of antigens responsible for IgG responses were plotted for each macaque over the course of 257 days for intravaginally and 272 days for intrarectally SIV_MAC_251 infected macaques. Each bar represents breadth (the number of antigens (Env, Gag, Tat and/or Nef) positive in any combination for IgG responses) at that specific time point. Absence of any bar represents no antigen-specific IgG responses detected for that time point. **b** Pearson coefficient of determination analysis between plasma viral load and breadth of IgG responses were shown for all macaques following SIV_MAC_251 inoculation for all time points. Note, GN91 had significant correlation values that were shown in bold numbers. Animal AE14 had no detectable IgG responses upto 236dpi against any SIV antigen and therefore no value of correlation was determined. R and P denote Pearson R and probability values respectively for each animal when correlated with plasma viral load. *P* value <0.05 was considered significant

Similar to WVL-specific immunoglobulin responses, peptide-specific immunoglobulin responses measured by cumulative OD values were also correlated with pVLs for all RMs to determine if any of the peptide-specific responses may represent a key predictor for the control of plasma viremia. Cumulative IgG responses against Env, Gag, Nef and Tat linear peptides show statistically significant negative correlations in eight, four, three and one RM respectively (Fig. 1b). RM GN91 maintained pVL to approximately 10^3^ RNA copies/ml, where Env, Gag, Nef and Tat specific IgG responses significantly upregulated, which might be playing a key role in controlling pVL. Significant negative correlations between Env-specific IgG responses and pVL in three disease progressing RMs (CL86, DE50 and FK88, Table 1) suggested that single antigen-specific responses were not adequate to control pVL and/or the disease progression and a combination of different peptide-specific IgG responses might be important in controlling pVL. Despite several significant negative correlations detected between antigen-specific IgM and IgA responses and pVLs, these independent responses did not predict the disease outcome (Fig. 1b). The breadth of IgG responses (IgG responses against antigens like Env, Gag, Nef and/or Tat) were also correlated with pVLs in all animals (Fig. 3). One (GN91) out of total 13 RMs had significant negative correlation between pVL and the number of antigen responses (*r* = −0.64, p-value = 0.035; Fig. 3b), which suggested that breadth of antibody responses had an impact in controlling pVL. Infact, three (Env, Gag and Nef) antigen-specific IgG responses in GN91 were detected as early as 55dpi and by 126dpi IgG responses were prevalently detected against all four antigens (Env, Gag, Nef and Tat) and remained high throughout the time point of this study (Fig. 3a). No other animals had such early and increased breadth of IgG responses detected in this study. The breadth of IgG responses was evident more in Ivag compared to IR infected RMs where all Ivag animals had two or more antigen-specific IgG responses detected (Fig. 3a). AE14 RM from IR group had shown no detectable antigen-specific responses upto 236dpi. Another two RMs (N107 and P205) from IR group had shown selective Env-specific IgG responses throughout the study period. No significant correlation was detected between breadth of IgM or IgA responses and pVLs in any of these animals.

### Immunoglobulin classes share similar functional linear B-cell epitopes

**Fig. 4:**
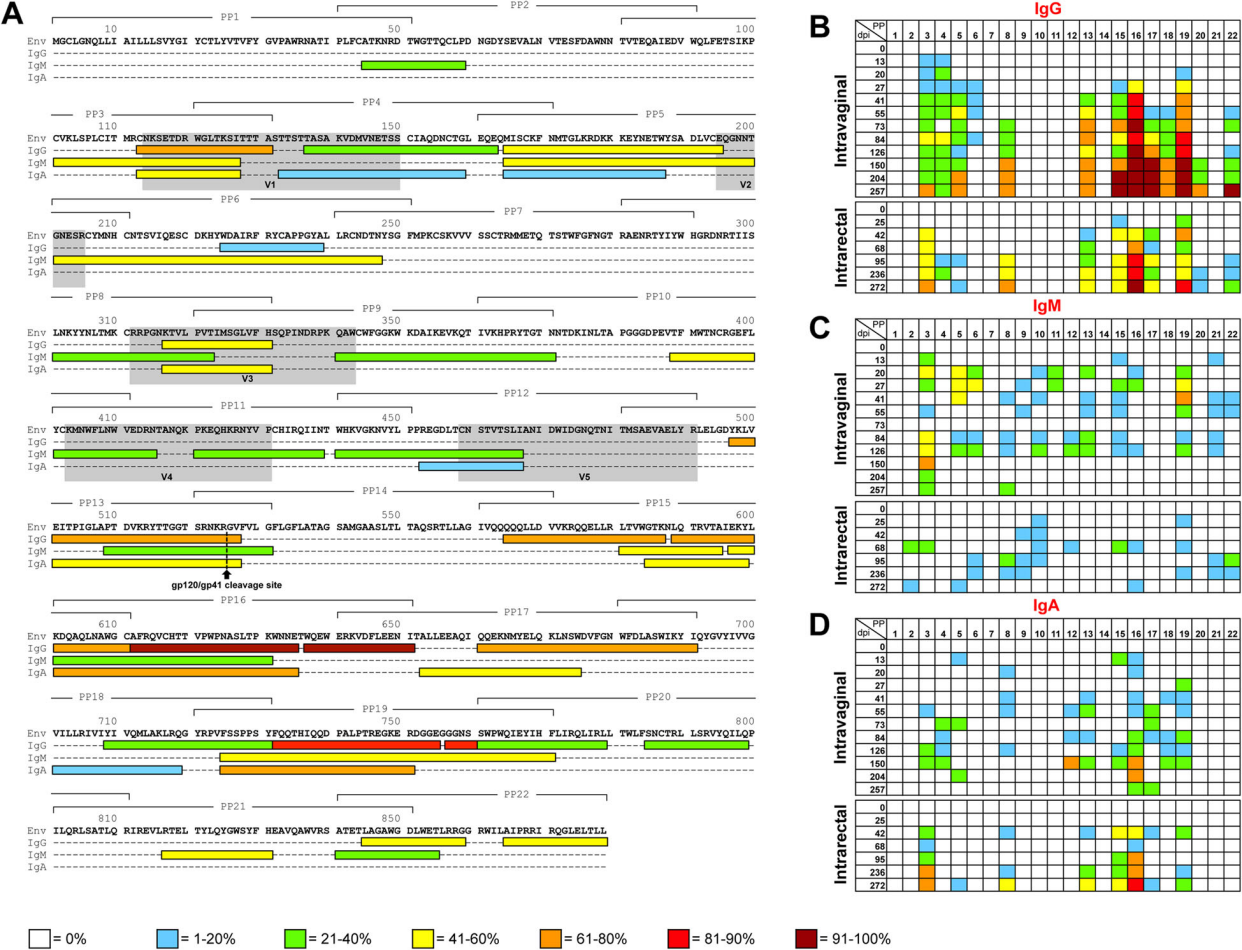
Antigen-specific immunoglobulin responses to linear SIV_MAC_239 Envelope peptides. **a** Amino acid sequence of SIV_MAC_239 Envelope protein, indicating regions containing potential linear epitopes responsible for peptide-specific IgG, IgA, and IgM responses were shown. Bracketed regions identify the amino acid sequences used to construct 15mer 10 peptide pools (PP 1–22). Colors refer to the percentage of animals exhibiting peptide-specific immunoglobulins for that region during infection with SIV_MAC_251 as shown in the key. Shaded regions show the variable regions V1-5, and the arrow indicates the proteolytic cleavage site for surface protein gp120 and transmembrane protein gp41. The percentage of macaques with peptide-specific IgG (**b**), IgM (**c**) and IgA (**d**) responses for SIV-Env peptides are shown for SIV infected macaques inoculated either intravaginally (*n* = 6) or intrarectally (*n* = 7). Responses to individual peptide pools (PP) are shown for each time point (days post infection; dpi). Colors refer to the percentage of animals exhibiting peptide-specific immunoglobulin responses for that region during infection with SIV_MAC_251 as shown in the key

**Fig. 5:**
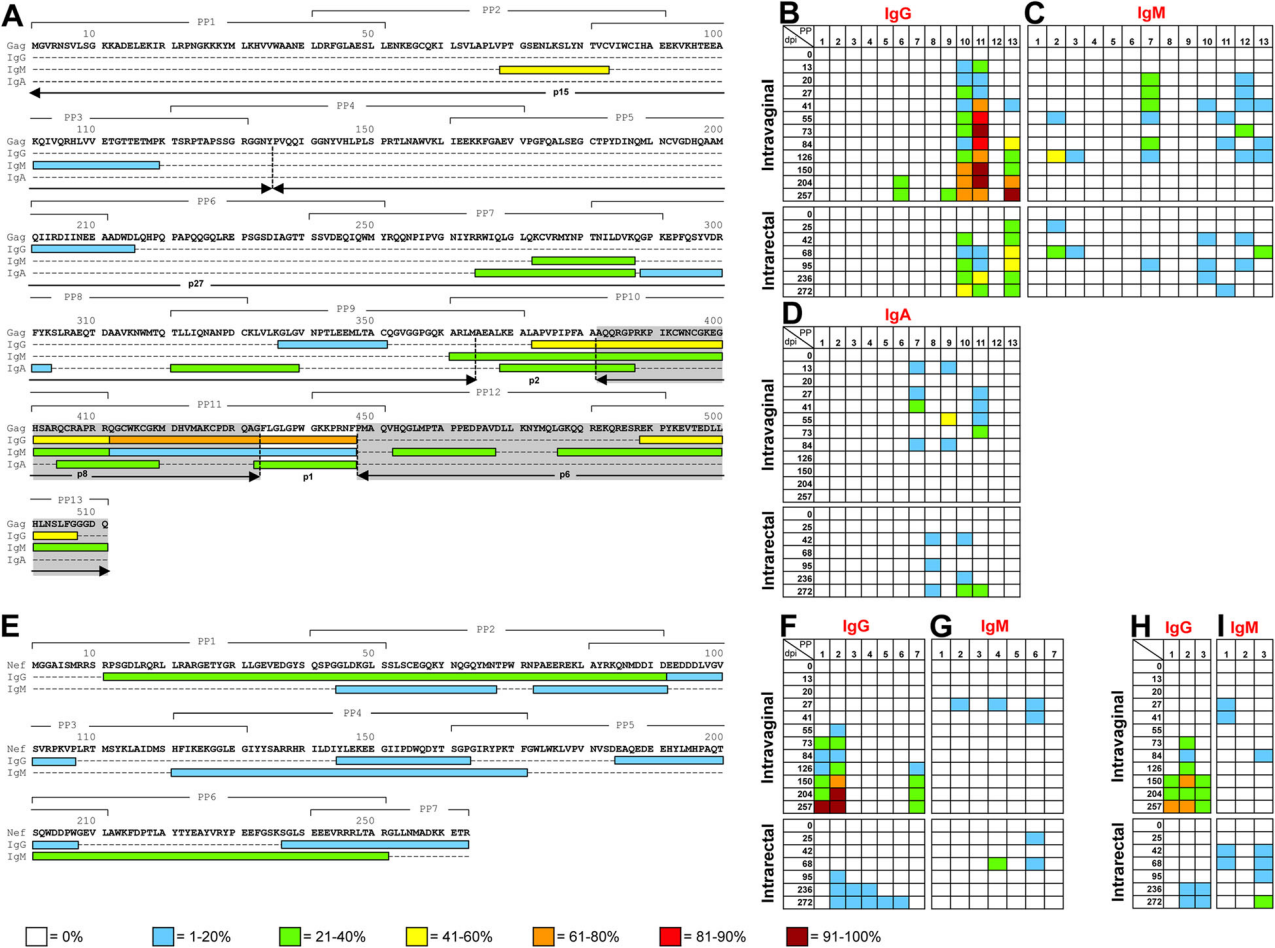
Antigen-specific immunoglobulin responses to linear SIV_MAC_239 Gag, Nef and Tat peptides. Amino acid sequences of SIV_MAC_239 Gag (**a**) and Nef (**e**) proteins, indicating regions containing potential linear epitopes responsible for peptide-specific IgG, IgM and/or IgA responses were shown. Bracketed regions identify the amino acid sequences used to construct 15mer 10 peptide pools (PP1-13 for Gag and PP1-7 for Nef proteins). Colors refer to the percentage of animals exhibiting peptide-specific immunoglobulins for that region during infection with SIV_MAC_251 as shown in the key. The Gag amino acid sequence (**a**) was divided into 6 proteins (p15, p27, p2, p8, p1 and p6) with two important regions, p8 (nucleocapsid region) and p6, shown in shaded area. The percentage of macaques with SIV-Gag peptide-specific IgG (**b**), IgM (**c**) and IgA (**d**) responses, SIV-Nef peptide-specific IgG (**f**) and IgM (**g**) and SIV-Tat peptide-specific IgG (**h**) and IgM (**i**) responses were shown for SIV infected macaques inoculated either intravaginally (*n* = 6) or intrarectally (*n* = 7). Responses to individual peptide pools (PP) were shown for each time point (days post infection; dpi). Colors refer to the percentage of animals exhibiting peptide-specific immunoglobulin responses for that region during infection with SIV_MAC_251 as shown in the key. Note that Nef- and Tat-specific IgA responses were not detected

Individual 15-mer peptides (11-aa overlap) for SIV-Env, Gag and Nef antigens were used to identify functional linear B-cell epitopes using antigen specific ELISA protocol mentioned above (Figs. 4 and 5). In general, peptide-specific IgG responses were detected against multiple regions of all proteins and increased following SIV infection in both Ivag and IR inoculated macaques (Figs. 4b and 5b, f). By 73dpi all Ivag infected RMs responded to both SIV-Gag and Env PPs with 100% responding to Env PP16 and 19 and Gag PP11 (Figs. 4b and 5b). Conversely, some macaques infected via the IR route did not respond to any PPs until 272dpi, when 100% of animals responded to Env PP16 (Figs. 4b and 5b). Peptide-specific IgM responses in Ivag infected macaques appeared during early infection and diminished overtime (Figs. 4c and 5c, g, i). Peptide-specific IgA responses were limited and inconsistent compared to peptide-specific IgG responses in Ivag and IR infected macaques and there were no detectable Nef or Tat-specific IgA responses (Figs. 4d and 5d). SIV infection via different mucosal routes appears to induce similar systemic antigen-specific immunoglobulin responses implying vaccine targets should be similar, regardless of the route of mucosal transmission.

Overall, the majority of the three immunoglobulin classes bound the same regions of Env peptides (e.g., first 31-aa in PP16, Fig. 4a). High Ab binding was also observed for Env-PP13, V1-V3 regions of gp120, and gp41. During SIV infection, the majority of the Env-specific IgG and IgA responses were generated against gp41, however, this was not statistically significant for either route of inoculation when compared to the binding with gp120 (Fig. 4b, *p* > 0.05). V2-V3 of gp120 played an important role in inducing antigen-specific IgM responses.

Gag-specific (p15 and p27) immunoglobulin responses were limited, and most Ab responses occurred in the p2, p1 and highly conserved p8 and p6 regions (Fig. 5a). PP11 had important B-cell epitopes that bound to IgG, IgA and IgM Abs (Fig. 5a). The Gag-specific IgM responses were similar to that of IgG and IgA, where a moderately strong affinity for the Gag-PP7 region was detected during acute infection, however this response diminished in chronic infection (Figs. 5c & d).

In the IR inoculated SIV-infected RMs, Nef-specific IgG responses were limited to BG21 and appeared late during infection (95dpi). However, Nef-specific IgG responses were detected early at 55dpi only in GN91, a RM from Ivag inoculated SIV-infected group. Moreover, Ivag inoculated SIV-infected RMs that survived upto 257dpi exhibited Nef-specific IgG responses at late chronic phase of infection (Fig. 5f). SIV-Nef epitopes varied between IgG and IgM and were scattered throughout the Nef protein (Fig. 5e).

Tat-specific responses appeared as early as 73dpi in Ivag infected macaques compared to IR infected macaques where the responses were detected at 236dpi. GN91 was one of the several animals with strong responses to this antigen; however, it showed no differences in the early generation of these responses when compared to other animals.

### Functional linear Envelope B-cell epitopes are 12 amino acid in length

SIV-Envelope epitope mapping was performed with plasma collected from 4 chronically Ivag SIV-infected macaques (BC35, CL86, CL87 and GN91, more than 150dpi) using the antigen-specific IgG ELISA protocol as described above. Five to 12-aa peptides from a 15mer SIV-Env peptide (sequence: KTVLPVTIMSGLVFH in V3 region) were synthesized commercially (GenScript, USA) and used as coating antigen at the concentration of 5 μg/ml to determine the exact length of a linear B-cell epitope. ELISA cutoff values were determined from SIV naïve macaques as described above. Positive IgG responses were detected when peptides with 12aa or greater in length were tested, which suggests that the minimum size of the functional linear epitope for eliciting antibodies was 12aa in length (Table 3). Individual 15-mer SIV-Env antigen was also used to determine the length of B-cell epitope responsible for binding responses (Fig. 4a).

**Table 3:**
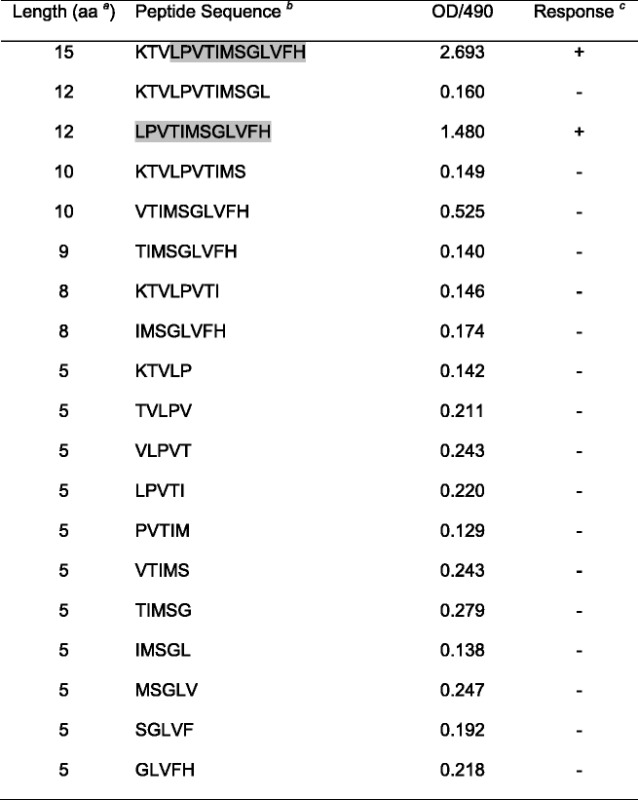
Determination of SIV-specific B-cell epitope length by ELISA

^a^aa denotes amino acid

^b^Peptides created based upon sequence of SIV_MAC_239 Envelope peptide (KTVLPVTIMSGLVFH)

^c^“+” denotes positive response determined by OD/490 ≥ 0.520 for *n* = 4

Note: Grey shaded area represents the functional linear B-cell epitope (12aa in length) that are binding to plasma IgG in SIV infected animals

## Discussion

Identification of broadly neutralizing antibodies (bNAbs) directed at the Env protein provides a possible solution to generate antibody based vaccine strategies. However, all HIV vaccines assessed to date were unable to generate bNAbs. The contribution of bNAbs to the control of early HIV-1 infection remains uncertain, as bNAbs develop relatively late in infection. Approximately 10–25% of HIV-1 infected people develop bNAbs within 3-years post infection [33]. Moreover, SIV_MAC_251 and SIV_MAC_239 were shown to be relatively resistant to antibody-mediated neutralization by both autologous and MAbs treatment [34, 35]. In our earlier study with either SHIV immunized or naïve SIV_MAC_251 challenged macaques, none of the macaques were able to generate significant levels of neutralizing Abs against either pathogenic SIV_MAC_251 or laboratory-adapted SIV_MAC_251 [23]. In this study, the presence of NAbs did not predict the outcome of the disease and moreover the occurrence of those antibodies might be too late to control the infection at that point.

GN91 was able to control pVL to approximately 1 x 10^3^ RNA copies/ml of plasma and had significantly upregulated WVL-specific IgG and IgM responses compared to CL87 suggesting that binding Ab responses might be playing an important role in clearing pVL. Our overall data implies that combination of different peptide-specific IgG responses are predictive of the control of plasma viremia compared to the peptide-specific IgM and IgA responses. Our findings corroborate prior studies showing that while gp41 and gp120 show high levels of binding to IgG, there appears to be no correlation with control of early viral load, and subsequent Env-specific IgG responses had little impact on disease progression [36–38] when present without additional responses. Gag has been shown to have little antiviral function [39], however, it appears to be a strong target for antigen-specific immunoglobulin responses. The majority of immunoglobulins bind around the conserved NC, p8, and p6 regions of the Gag protein, which have several important functions in viral pathogenesis, such as viral replication, encapsulating the viral genome, aiding in reverse transcription, viral genome packaging, and viral budding [40]. While Gag is not a target for bNAbs, B-cell recognition of these highly important and conserved regions of the Gag protein may be indicative of a potential target for vaccine design. Limited data are available on the role of Nef-specific Abs in HIV disease progression. Absence of anti-Nef Abs was found to be associated with symptomatic HIV infection [41]. In our study, we identified increased and early Nef-specific IgG responses in GN91, which controlled pVL much earlier than other disease progressing RMs. Our findings, along with the recent single domain antibody study for the inhibition of Nef protein in a mouse model [42], indicate that Nef needs to be considered as an important target for a novel therapeutic approach in the prevention and control of HIV. Several studies demonstrated that Tat-specific antibodies are more common in individuals who are successfully controlling the disease, suggesting that Tat-specific Abs have a beneficial role in preventing disease progression [43–48]. In this study, we detected the presence of Tat-specific IgG in several animals, but as we have shown, it appears to be part of a large overall antigen-specific response that plays role in controlling pVL. Early induction of IgG responses against important targets and maintenance of those responses at higher magnitude might be crucial in reducing plasma viral loads and a possible predictor of disease outcome.

We also asked whether linear epitopes for antigen-specific immunoglobulins identified in this study corresponded to bNAb targets. The b12 bNAb recognizes almost exactly the same region as CD4, where these conserved regions of the CD4bs [49] fall within Env-PP10 and PP11 and induced very limited peptide-specific IgM responses (Fig. 4). However, the CD4bs is highly conformational, whereas only linear epitopes were examined in this study. Similarly, three series of bNAbs, specifically PG9, PG16 [50], and CH01-CH04 [51] target conserved conformational regions within the variable loops (V1-V3) of gp120. While these interactions have been shown to be highly conformational, the analogous V1-V3 regions in SIV_MAC_239 were shown to be strong targets for peptide-specific IgG, IgA and IgM responses in SIV infected macaques (Fig. 4). Two bNAbs, 2F5 and 4E10, target the highly conserved MPER region of gp41 [52]. Linear epitopes for 4E10 and 2F5 on HIV-1 strain HXB2 represent NWFNIT and ELDKWA peptide sequences respectively [52], and these regions correspond to PP17 region in SIV_MAC_239 (665–678aa). Seventy percent of SIV-infected macaques generated antigen-specific IgG responses to this region, and several macaques also generated antigen-specific IgA responses to this region. Therefore, Env-specific immunoglobulins bind to several linear epitopes of recently identified bNAbs, with the exception of the CD4bs. In future studies, it will be important to define the plasma viral sequence data in SIV infected animals and determine if the presence of escape variant(s) have any correlation with the antigen-specific immunoglobulin responses.

In summary, quantitative and qualitative immunoglobulin responses were detected in SIV-infected macaques, where increased IgG responses were measured specifically against the gp41 region followed by variable regions V1-V3 of gp120, and NC and p6 region of Gag protein. Antigen-specific IgM and IgA responses were more limited but targeted towards similar regions of the Env and Gag proteins. Several regions in Env protein strongly bind to immunoglobulins and are important epitopes for bNAbs. Early induction and increased breadth of antigen-specific IgG responses might be crucial to the control of plasma viral load and predictive of disease progression in SIV/HIV infection.

## Conclusions

Our data strongly suggest that an early induction and increased breadth of peptide-specific IgG responses are indicative of disease progression. While Gag-specific responses may be argued to be an indirect indicator of HIV disease progression, recent identification of Abs against Nef and Tat proteins suggests that it may be possible to prevent and control HIV/AIDS by targeting the function of Tat and Nef proteins. Moreover, the presence of NAbs did not predict the outcome of the disease in our study where the generation of those antibodies might be too late to control the SIV infection. The experimental design does not address the role of T-cell responses and other innate immune responses in regulating plasma viral load in these SIV-infected animals.

## Additional file

Additional file 1: Table S1. Quantification of total IgG, IgM and IgA following SIV_MAC_251 infection in rhesus macaques (PDF 2059 kb)
