# Supplementary material for: Breadth and magnitude of antigen-specific antibody responses in the control of plasma viremia in simian immunodeficiency virus infected macaques
Source: Virol J. 2016 Dec 1;13:200. doi: 10.1186/s12985-016-0652-x (PMC5131515; doi:10.1186/s12985-016-0652-x)
Supplement: Additional file 1: Table S1. — Quantification of total IgG, IgM and IgA following SIVMAC251 infection in rhesus macaques (PDF 2059 kb) [file 12985_2016_652_MOESM1_ESM.pdf]

Additional file 1: Table S1. Quantification of total IgG, IgM and IgA following $SIV_{MAC}251$ infection in rhesus macaques

Intravaginally infected animals

Total IgG (μg/ml of plasma)

Days post infection	BC35	CL87	GN91	CL86	DE50	FK88	Mean Value
**0**	1954	6894	8735	3102	2209	3461	4393
**13**	1573	5581	3394	2517	2121	2751	2990
**20**	5664	4363	9234	2203	1780	5728	4829
**27**	7444	6600	8578	4625	4369	6531	6358
**41**	4144	7360	2240	3396	6448	5560	4858
**55**	4303	3810	4015	2904	9199	6439	5112
**73**	5344	4026	5973	-	-	-	5115
**84**	7589	5131	2988	2547	7810	2153	4703
**126**	8576	9932	6357	5389	13978	3093	7887
**150**	4888	4049	6752	-	-	-	5230
**204**	11359	9019	6639	-	-	-	9006
**257**	10340	10828	6932	-	-	-	9367

Total IgM (μg/ml of plasma)

Days post infection	BC35	CL87	GN91	CL86	DE50	FK88	Mean Value
**0**	1726	1850	1292	1123	2739	679	1568
**13**	2213	1482	174	1554	1112	1128	1277
**20**	903	2540	144	4147	914	1327	1662
**27**	1211	734	398	1122	1623	1272	1060
**41**	939	1715	157	794	3681	1034	1387
**55**	1190	2947	203	1455	3117	407	1553
**73**	1629	1649	1604	-	-	-	1627
**84**	1987	1554	893	1966	3911	155	1744
**126**	1866	2054	731	3181	2595	234	1777
**150**	1513	389	416	-	-	-	772
**204**	719	860	71	-	-	-	550
**257**	1063	1010	2697	-	-	-	1590

Total IgA (μg/ml of plasma)

Days post infection	BC35	CL87	GN91	CL86	DE50	FK88	Mean Value
**0**	1200	1181	720	365	2546	314	1054
**13**	1117	1426	725	502	1288	198	876
**20**	843	1363	832	620	1265	1135	1010
**27**	749	375	416	235	2769	1135	946
**41**	948	640	447	335	690	1110	695
**55**	1157	841	892	414	1189	1431	987
**73**	1069	1683	1414	-	-	-	1388
**84**	1573	1931	887	1434	1063	908	1299
**126**	1145	2258	497	2019	441	921	1213
**150**	1589	896	288	-	-	-	924
**204**	1371	738	204	-	-	-	771
**257**	757	804	336	-	-	-	632

Intrarectally infected animals

Total IgG (μg/ml of plasma)

Days post infection	AE14	AP09	AP64	BG21	N107	P205	T153	Mean Value
0	1918	929	1411	1249	1149	1908	2164	1533
25	1786	1906	1546	2089	1158	2025	2717	1889
42	2317	2099	1773	2759	1173	1979	2677	2111
68	2883	1039	2244	1982	1390	1547	3237	2046
95	1809	1078	1793	2087	1065	1587	3538	1851
236	1147	1331	2899	4582	902	898	3356	2159
272	5139	3518	3377	5711	960	-	5463	4028

Total IgM (μg/ml of plasma)

Days post infection	AE14	AP09	AP64	BG21	N107	P205	T153	Mean Value
0	1184	1081	248	1607	3227	2173	2123	1663
25	997	2092	836	1725	2576	2037	3112	1911
42	3129	2769	1669	1050	3140	7209	4787	3393
68	3727	2008	812	2377	3733	2362	2112	2447
95	2368	1756	850	1275	3360	2746	4286	2377
236	1494	3402	509	1397	2487	2029	1046	1766
272	1796	2319	509	1222	2081	-	1090	1503

Total IgA (μg/ml of plasma)

Days post infection	AE14	AP09	AP64	BG21	N107	P205	T153	Mean Value
0	1919	309	203	2031	161	906	592	874
25	1496	393	205	2031	117	727	615	798
68	2764	345	154	2089	121	1433	744	1093
236	1277	364	159	1926	99	814	1187	832

Note: “-” denotes no data recorded for that time point
